# Evidence of much wider distribution of the potential West Nile virus vector, *Culex modestus*, in the UK

**DOI:** 10.1186/s13071-025-06936-3

**Published:** 2025-11-24

**Authors:** Anthony J. Abbott, Bathsheba L. Gardner, Harrison Hardy, Alexander G. C. Vaux, Colin J. Johnston, Roksana Wilson, Amy C. Edwards, Jonathan Yardley, Arran J. Folly, Jolyon M. Medlock

**Affiliations:** 1 Medical Entomology & Zoonoses Ecology (MEZE), Centre for Climate and Health Security, UK Health Security Agency, Porton Down, Salisbury, SP4 0JG UK; 2 Animal and Plant Health Agency, Woodland Lane, Addlestone, KT15 3NB UK

**Keywords:** *Culex modestus*, West Nile virus, Usutu, United Kingdom, Adult trapping, Larval sampling

## Abstract

*Culex modestus* is a primary vector of West Nile virus (WNV) in Europe, feeding on both avian and mammalian hosts. Since its rediscovery in the UK in Kent in 2010, its range has been reported throughout the coastal parts of the Thames Estuary, as well as parts of Suffolk and Cambridgeshire. This paper reports further detections of *Cx. modestus* in the UK in new localities. Surveillance was conducted using Mosquito Magnet and BG-Pro traps baited with synthetic lures between June and October 2024, supplemented by field sampling for immature stages. Populations were detected at two new localities in Cambridgeshire (Ouse Fen, Fowlmere) and on the Sussex/Hampshire border (Pagham Harbour, Farlington Marshes), with high abundances in some localities. The presence of *Cx. modestus* at new locations suggests ongoing range expansion in the UK. Continued monitoring of this mosquito is important for determining and managing potential WNV transmission risk to public health.

**Graphical Abstract:**

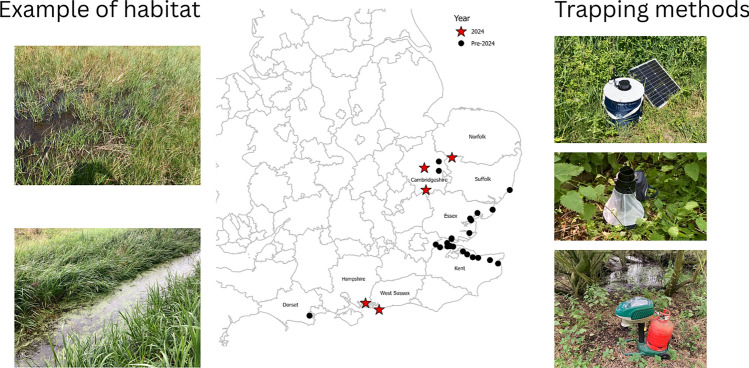

Owing to its ability to feed on both avian and mammalian host species, the mosquito *Culex* (*Barraudius*) *modestus* (Ficalbi, 1890) is an important disease vector and a principal bridge vector for West Nile virus (WNV) [1, 2], having been implicated in outbreaks in both humans and equids in Europe [3–5]. It is also believed to be an important vector for Usutu virus (USUV), with RNA detected in field-caught *Cx. modestus* in the Czech Republic [6, 7], and vector competence studies in Belgium showing a high transmission potential [8]. It was first identified in the UK in 1944 in Hayling Island [9]; however, despite sampling in the area, it was not found again until 2010 in North Kent [10]. Since its rediscovery, the known range of *Cx. modestus* has increased to include various sites in coastal and estuarine Essex and a few localities in Cambridgeshire [11, 12], and recently, Suffolk [13].

USUV has been detected in the UK in birds [14] and mosquitoes [15], and more recently, WNV has been detected in UK mosquitoes [16]. Research has shown that WNV and USUV are closely related [17], and they are both members of the Japanese encephalitis virus (JEV) serocomplex [18]. There is evidence suggesting that USUV emergence precedes WNV, with cases of USUV being reported prior to those of WNV in Austria [19], Germany [20], Switzerland [21], and the Netherlands [22]. USUV and WNV co-circulate in many European countries [23]. During 2024, 19 European countries reported WNV cases, including France and Germany [24], with 2481 cases reported in humans.

This article reports new findings of *Cx. modestus* in the UK, found as a result of mosquito surveillance carried out by the Medical Entomology and Zoonoses Ecology group, UK Health Security Agency. This includes the long-term Nationwide Mosquito Programme, which has run annually since 2010; Mosquito Snapshot, a new project run for the first time in 2024; and as part of the UKRI funded VB-RADAR project, a collaboration with the Animal and Plant Health Agency (APHA), Zoological Society of London (ZSL), and British Trust for Ornithology (BTO), since 2023.

Adult trapping using a Mosquito Magnet trap (Mosquito Magnet^®^ Executive model, Woodstream Corporation, St. Joseph, MO, USA) baited with an octenol lure (1-octen-3-ol) was conducted fortnightly between June and October 2024 in 38 wetland sites across England. In each trapping week, traps were operated continuously over four trap nights, Monday to Friday. In addition, 72 BG-Pro traps (baited with BG-Lure and BG-yeast; Biogents AG Regensburg, Germany) were deployed in each 50 km grid square in England on two occasions during the summer (three continuous trap nights each during the periods 12–25 August and 2–15 September). In addition, five BG-Sentinel 2 traps, baited with BG-Lure, were operated in five wetlands in Cambridgeshire between June and late September, operated weekly, with samples collected every 7 days.

Sampling for larvae and pupae was conducted in Cambridgeshire, Suffolk, West Sussex, and Hampshire at sites nearby or where new records of *Cx. modestus* were collected in adult traps. Larval sampling was performed using a 200 ml dipper and targeting suitable aquatic habitats, such as wet grassland, permanent ditches, and reedbeds. Dipping was performed ad hoc with sampling taking place at suitable water bodies that were accessible and in aquatic habitats similar to those identified in previous studies [13].

*Culex modestus* was detected at the following new wetland locations in 2024 (Table 1; Fig. 1): (A) 233 adult female *Cx. modestus* were trapped in a Mosquito Magnet trap at Ouse Fen, in addition to 27 larvae; (B) a single female *Cx. modestus* in a BG-Sentinel 2 trap at Fowlmere, Cambridgeshire, with no larvae detected during field sampling; (C) 34 female *Cx. modestus* at Wicken Fen in July and August, where two larvae had previously been found [11]. A single larva was also recorded at Wicken Fen. (D) A single *Cx. modestus* larva was found in Lakenheath Fen in week 35, although no adults of this species were found in the Mosquito Magnet trap placed nearby (June–November). No *Cx. modestus* were trapped using adult traps at other sites in the Cambridgeshire Fens: Welney, Chippenham Fen, Kingfisher Bridge, and Ouse Washes. Field sampling for immatures during week 38 found no evidence for larval *Cx. modestus* at Ouse Washes, Welney, or Woodwalton.

**Table 1:** Location and numbers of adult and immature *Cx. modestus* collected from new sites during 2024

Site	Latitude/longitude	Adults	Larvae
Ouse Fen	52.3342, 0.0157	12♀ in week 27; 194♀ in week 33; 27♀ in week 35	27 in week 38
Fowlmere	52.0921, 0.0499	1♀ in week 33	0
Wicken Fen	52.3116, 0.2889	5♀ in week 30; 15♀ in week 31; 12♀ in week 33; 2♀ in week 24	1 in week 36
Lakenheath Fen	52.4488, 0.5191	0	1 in week 35
Welney	52.5269, 0.2765	Absent	Absent
Chippenham Fen	52.2961, 0.4200	Absent	Absent
Kingfisher Bridge	52.3372, 0.2651	Absent	Absent
Ouse Washes	52.4515, 0.1619	Absent	Absent
Woodwalton Fen	52.4478, -0.1883	NA	Absent
Pagham Harbour	50.7599, -0.7872	6♀ in week 31, 4♀ in week 37	25 in week 32
Farlington Marshes	50.8339, -1.0283	5♀ in week 35	3 pupae in week 38

**Fig. 1:**
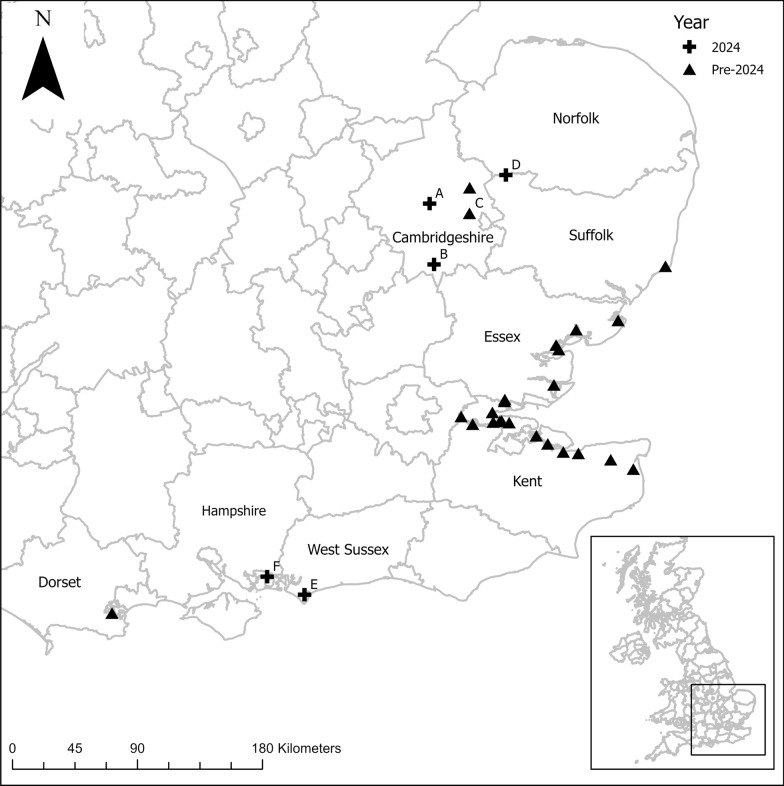
Known locations for *Cx. modestus* in southern England. Black crosses indicate the location of new records in 2024. Black triangles indicate known locations since 2010. The insert map shows the location of study areas in the UK

On the south coast of England, (E) ten adult female *Cx. modestus* were trapped in a Mosquito Magnet trap at Pagham Harbour, and 25 larvae collected from wet grassland. (F) Five female *Cx. modestus* were trapped at Farlington Marshes, Hampshire, with the subsequent detection of three pupae collected in wet grassland and reared through to the adult stage.

The findings at Pagham Harbour (West Sussex) and Farlington Marshes (Hampshire) are the first records in this area since the 1940s and may represent a range expansion. However, there has been little targeted adult or larval surveillance in this region except in recent years at Hayling Island, due to nuisance from other species. There are previously published records from the region, with two adult females on Hayling Island (July 1944) and six larvae from water tanks in Portsmouth (August 1944), followed by an adult in Hayling Island (May 1945) and larvae collected in Gosport (June 1945), though, this was not believed to be representative of an established population [9], so this could be evidence of a recrudescence of a long assumed extinct population. The closest recent record on the south coast of England is in Poole Harbour at Arne, Dorset, where one adult female was recorded at a Mosquito Magnet trap, but no larvae could be found during surveys for immatures [11]. In other more widely distributed locations on the south coast, trapping has been conducted in the vicinity at Southampton port (2010), Brownsea Island (Dorset; 2011 and 2012), Hayling Island (Hampshire; 2014, 2015, and 2016), Langley (Hampshire; 2016), Ringwood (Hampshire; 2017, and 2018), Poole (Dorset; 2018), and Radipole, near Weymouth (Dorset; 2018) as part of nationwide mosquito surveillance. During these surveys, no *Cx. modestus* were ever detected [13]. The absence of any *Cx. modestus* at Hayling Island would generally suggest that the species was not present and that *Cx. modestus* had not established itself in this locality since the 1940s. The nearest recent finding in Arne was found as a single adult in 2011 [11], with none detected in subsequent immature sampling, or adult sampling in 2012, 2013, 2016, and 2017 [13]. However, these recent findings cast some doubt on this, and further surveys are needed.

The records at Ouse Fen and Fowlmere are new locations in Cambridgeshire, though they remain close to where *Cx. modestus* has previously been found in Ely [12]. The record at Wicken Fen confirms the presence of *Cx. modestus* here, having previously only been found as immatures more than a decade ago [11]. The large number of adults found at Ouse Fen indicates *Cx. modestus* is established and abundant at the site. The finding of a single larva at Lakenheath Fen, represents the northernmost record of *Cx. modestus* in the UK to date. Further surveillance will continue to focus on wetlands in southeast England, along the south coast, as well as in the Lincolnshire Fens, Romney Marshes, and Norfolk Broads to identify any additional locations *Cx. modestus* may be present. The recent evidence of WNV detected in UK mosquitoes highlights the importance of generating data on the presence of this competent bridge vector. An updated map of their distribution, along with details of its ecology, is critical for WNV outbreak risk assessment, preparedness, and response.
